# SIRT6 Inhibitor, OSS_128167 Restricts Hepatitis B Virus Transcription and Replication Through Targeting Transcription Factor Peroxisome Proliferator-Activated Receptors α

**DOI:** 10.3389/fphar.2019.01270

**Published:** 2019-10-25

**Authors:** Hui Jiang, Sheng-Tao Cheng, Ji-Hua Ren, Fang Ren, Hai-Bo Yu, Qing Wang, Ai-Long Huang, Juan Chen

**Affiliations:** The Key Laboratory of Molecular Biology of Infectious Diseases designated by the Chinese Ministry of Education, Department of Infectious Diseases, Institute for Viral Hepatitis, The Second Affiliated Hospital of Chongqing Medical University, Chongqing Medical University, Chongqing, China

**Keywords:** OSS_128167, SIRT6, hepatitis B virus, antiviral, core promoter, peroxisome proliferator-activated receptors α

## Abstract

Hepatitis B virus (HBV) is a major public health threat and anti-HBV drugs are limited to nucleos(t)ide analogs (NAs) and pegylated interferon alpha (Peg-IFNα). Toward identifying an effective compound for HBV treatment is important to suppress and eradicate HBV. In this study, we explored the anti-viral effect of Sirtuin 6 (SIRT6) inhibitor, OSS_128167, in HBV transcription and replication. Firstly, we found that OSS_128167 could decrease the level of HBV core deoxyribonucleic acid (DNA) and 3.5-Kb ribonucleic acid (RNA) *in vitro*. Furthermore, the level of HBV DNA and 3.5-Kb RNA were also markedly suppressed by OSS_128167 administration in HBV transgenic mice. In addition, we found that depletion of SIRT6 inhibited HBV transcription and replication in HepG2.2.15 and HBV-infected HepG2-sodium taurocholate cotransporting polypeptide cells, whereas overexpression of SIRT6 enhanced HBV transcription and replication. Importantly, the positive effect of SIRT6 overexpression on HBV transcription could be blocked by OSS_128167 treatment. Further mechanism studies showed that HBV core promoter was significantly activated by SIRT6 through upregulating peroxisome proliferator-activated receptors α (PPARα) expression. And ectopical expression of SIRT6 or PPARα relieved the restriction of HBV transcription mediated by OSS_128167. In summary, our results showed that OSS_128167 might serve as a potential antiviral agent for HBV therapy and SIRT6 played a pivotal role in HBV transcription and replication.

## Introduction

Despite the effective vaccine is universally available, hepatitis B virus (HBV) infection is still a major public health problem and leading to end-stage liver diseases, such as hepatitis and hepatocellular carcinoma (Ott et al., 2012). HBV is a liver tropic virus with a 3.2-Kb partially double-stranded deoxyribonucleic acid (DNA) genome, termed relaxed circular DNA (rcDNA). Upon viral infection, nucleocapsid is released into cytoplasm, then transported to nucleus in which rcDNA is converted to covalently closed circular DNA (cccDNA). The cccDNA can serve as the template to generate HBV transcripts, including 3.5-Kb ribonucleic acid (RNA) (precore mRNA and pregenomic RNA), 2.4-Kb mRNA, 2.1-Kb mRNA, and 0.7-Kb mRNA (Shlomai et al., 2014). It is worth noting that 3.5-Kb RNA not only can work for translation template of secreted e antigen (HBeAg), core protein, and viral polymerase, but also can be packaged into nucleocapsid for reverse transcription. As the promoter of 3.5-Kb RNA, HBV core promoter plays an important role in HBV life cycle and inhibition of core promoter activity can restrict HBV transcription and replication dramatically (Ko et al., 2017). Therefore, transcription silence of HBV core promoter may represent an attractive approach for HBV therapy.

Current FDA approved therapeutic for chronic HBV infection (CHB) are limited to nucleos(t)ides (NAs) and interferon alfa (IFNα) (Ghany, 2017). NAs including lamivudine, adefovir, entecavir, tenofovir, and telbivudine decrease HBV DNA level markedly by inhibiting viral reverse transcription. However, long-term use of these NAs often results in generation of drug-resistant virus (Zhang et al., 2018). IFNα is showed to be useful in part of individuals, but has serious side effects (Kim et al., 2016). Therefore, identification of new agents will contribute to the development of curative therapies for CHB. To identify the effective molecular, we screened 3000 compounds from a small molecular compound library and focused on OSS-128167, a selective inhibitor of sirtuin 6 (SIRT6). SIRT6 is an NAD^+^-dependent deacetylase with a role in regulation of DNA repair, telomere maintenance, glucose and lipid metabolism, inflammation, and cancer (Kugel and Mostoslavsky, 2014). Therefore, SIRT6 inhibitor may serve as a potential therapeutic agent in diabetes, immune-mediated disorders, and cancer. It has reported that OSS-128167 exhibited increased sensitivity to melphalan and doxorubicin in primary multiple myeloma (MM) cells, as well as in melphalan-resistant (LR-5) and doxorubicin-resistant (Dox40) MM cell lines (Cea et al., 2016). However, the effect of OSS-128167 on HBV transcription and replication is not reported yet.

In this study, we revealed that SIRT6 inhibitor, OSS_128167, could restrict HBV transcription and replication *in vitro* and *in vivo*. Mechanically, SIRT6 enhanced HBV core promoter activity through upregulating PPARα expression. Notably, our data suggest that OSS_128167 may serve as a potent therapeutic for treatment of HBV and SIRT6 plays an important role in HBV infection.

## Materials and Methods

### Cell Culture

HepG2.2.15 cells were obtained from the Shanghai Second Military Medical University and was maintained in Dulbecco’s modified Eagle medium (DMEM) containing 10% fetal bovine serum (FBS) and 400 μg/ml G418. Huh7 and HepG2-NTCP cells were maintained in DMEM supplemented with 10% FBS, 100 IU/ml penicillin, and 100 μg/ml streptomycin. All cells were maintained in a humidified incubator at 37°C with 5% CO2. Cells applied for experiments were all within 10 generations.

### Western Blot

Cells were lysed in radioimmunoprecipitation assay buffer containing protein inhibitor at 4°C for 15 min, then the protein concentration was determined by bicinchoninic acid protein assay (Thermo scientific). Thirty micrograms of total protein was loaded and separated by SDS-PAGE, and then was transferred on polyvinylidene difluoride (PVDF) membrane (GE Healthcare). Membrane was blocked with 5% nonfat milk, followed by incubating with respective primary antibodies overnight at 4°C. Then the membrane was blocked with the second antibodies at room temperature for 2 h. Finally, protein was assayed by exposing on X-ray films.

### Ribonucleic Acid Extraction and Reverse Transcription

The RNA was extracted with TRIzol reagent (TianGen), and precipitated with isopropyl alcohol. Then RNA samples were further mixed with gDNA buffer to digest genome DNA, followed by reverse transcription with FastKing RT Kit (TianGen) to generate cDNA.

### Extraction of Hepatitis B Virus Core Deoxyribonucleic Acid

HBV core DNA was extracted as followed. Briefly, cells were lysed with 0.5 ml of lysis buffer (10 mM Tris-HCl pH 8.0, 1 mM EDTA, 1% NP-40, 2% sucrose) at 37°C for 15 min, then cell lysate was digested with 40 U/ml DNase (Takara) at 37°C for 4 h. Subsequently, virus particles were precipitated with 5% PEG8000 on ice for 1 h. Then precipitated virus particles were digested in the 0.5 ml proteinase K buffer containing 0.5 mg/ml proteinase K (Solarbio) overnight at 45°C. The core DNA was extracted with phenol chloroform twice and then precipitated with isopropyl alcohol. DNA samples were dissolved in double-stilled water.

### Hirt Extraction of Covalently Closed Circular DNA

The cccDNA was extracted by using modified Hirt extraction method. Briefly, the equal number of cells were lysed with 0.5 ml Hirt lysis buffer (1% SDS, 50 mM Tris-HCl PH8.0, 100 mM EDTA, 150 mM NaCl) at 37°C for 15 min. Then the cell lysate was mixed with 125 μl KCl (2.5 M) overnight at 4°C with gentle rotation. Cell debris was removed by centrifugation, and the cccDNA samples were extracted three times with phenol chloroform, followed by precipitating with isopropyl alcohol. Finally, the cccDNA was dissolved in the double stilled water. The extracted cccDNA was further subjected to T5 exonuclease treatment before cccDNA quantitative polymerase chain reaction (PCR).

### Quantitative Polymerase Chain Reaction

The level of HBV core DNA was determined by absolute quantitative PCR with FastStart Essential DNA Green Master (Roche). The specific primers were F: 5’-CCTAGTAGTCAGTTATGTCAAC-3’ and R: 5’-TCTATAAGCTGGAGGAGTGCGA-3.’ The quantitative PCR detection of cccDNA was performed with GoTaq Probe qPCR Master Mix (Promega). The selective primers and probe were F: 5’-CTCCCCGTCTGTGCCTTCT-3,’ R: 5’-GCCCCAAAGCCACCCAAG-3’ and probe: 5’-ACGTCGCATGGAGACCACCGTGAACGCC-3.’ The expression of target genes were detected by relative quantitative PCR with iTaq Universal SYBR Green Supermix (Bio-Rad), and the expression of β-actin mRNA was used as endogenous control. The primer sequences for: 3.5-Kb RNA (F: 5’-GCCTTAGAGTCTCCTGAGCA-3,’ R: 5’-GAGGGAGTTCTTCTTCTAGG-3’); total RNA (F: 5’-ACCGACCTTGAGGCATACTT-3,’ R: 5’-GCCTACAGCCTCCTAGTACA-3’); β-actin (F: 5’-CTCTTCCAGCCTTCCTTCCT-3,’ R: 5’-AGCACTGTGTTGGCGTACAG-3’).

### Southern Blot

HBV core DNA was extracted as described above. Then the DNA samples were electrophoresed on an agarose gel and followed by transferred on positive charged nylon membrane (Roche). After the prehybridization, the membrane was hybridized with specific probe overnight at 42°C with gentle rotation. After stringency washes, the membrane was incubated with Anti-Digoxigenin-AP Fab fragments (Roche) at 37°C for 30 min. Finally, the DNA level was assayed by exposing on an X-ray film.

### Immunofluorescence Assay

For the immunofluorescence assay, cells were plated on glass cover slips and stained as described below. Cells were fixed with 4% formaldehyde for 15 min and then incubated in Triton extraction buffer (300 mM sucrose, 20 mM HEPES pH7.9, 50 mM NaCl, 3 mM MgCl, 0.5% Triton X-100) for 30 min to increase the permeability of cell membrane. Next, the cells were blocked with 2% BSA for 40 min and incubated with rabbit anti-HBc polyclonal antibody (DAKO) at 4°C in wet box overnight. An FITC-conjugated anti-rabbit antibody was used as secondary antibody and DAPI was used to stain nuclei. Finally, confocal microscope was used to observe the images.

### Cell Viability Assay

(3-(4,5-dimethylthiazol-2-yl)-5-(3-carboxymethoxyphenyl)-2-(4-sulfophenyl)-2H-tetrazolium, inner salt) (MTS) assay (Promega) was used to assess cytotoxicity of OSS_128167 according to manufacturer’s instructions. Briefly, HepG2.2.15 and HepG2-sodium taurocholate cotransporting polypeptide (NTCP) cells were seeded on 96-well plate at a density of 2×10^4^ cells per well. After 24 h, the cells were treated with OSS_128167 at indicated concentrations. Seventy two hours later, 20 µl CellTiter 96^®^ AQueous One Solution Reagent was added to each well for 4 h at 37°C under 5% CO_2_. Finally, the absorbance at 490 nm was recorded by using a 96-well plate reader.

### Hepatitis B Virus Transgenic Mice

The HBV transgenic mice were obtained from Prof. Ningshao Xia (Xiamen University, China). These mice encode 1.2 over-length copy of HBV genome (ayw). All animals were kept under specific-pathogen-free condition. The male mice aged 6–8 weeks were divided into three groups (n = 8) randomly. The 50 mg/kg dose of OSS_128167 or vehicle were administered every 4 days by intraperitoneal injection. The entecavir (ETV) group (0.02 mg/kg) was administered every 4 days by oral gavage administration. At the indicated time points, the serum was collected through tail vein. And the mice were euthanized to collect liver tissues at 12 days post injection. The animal studies were in accordance with Chinese Council on Animal Care and approved by Chongqing Medical University.

### Immunohistochemistry

Immunohistochemical staining for intrahepatic core protein was performed on paraffin-embedded liver tissue sections as described. Briefly, formalin-fixed and paraffin-embedded specimens from mice liver were sectioned into 5-um-thick slices. After deparaffinization, the slides were incubated with rabbit anti-HBc polyclonal antibody (Dako) at 4°C overnight, and detected with diaminobenzidine (DAB) staining. Finally, the images were captured by microscope.

### Statistical Analysis

Data were expressed as the mean ± SD from three independent experiments. The comparison of mean between two groups was conducted by using Student-t test. The comparison for more than two groups was conducted using two-way ANOVA. A value of P < 0.05 was considered significant (*P < 0.05). All the statistical analyses were performed using the SPSS 19.0 software.

## Results

### Sirtuin 6 Inhibitor, OSS_128167, Inhibited Hepatitis B Virus Transcription and Replication *In Vitro*

**Figure 1:**
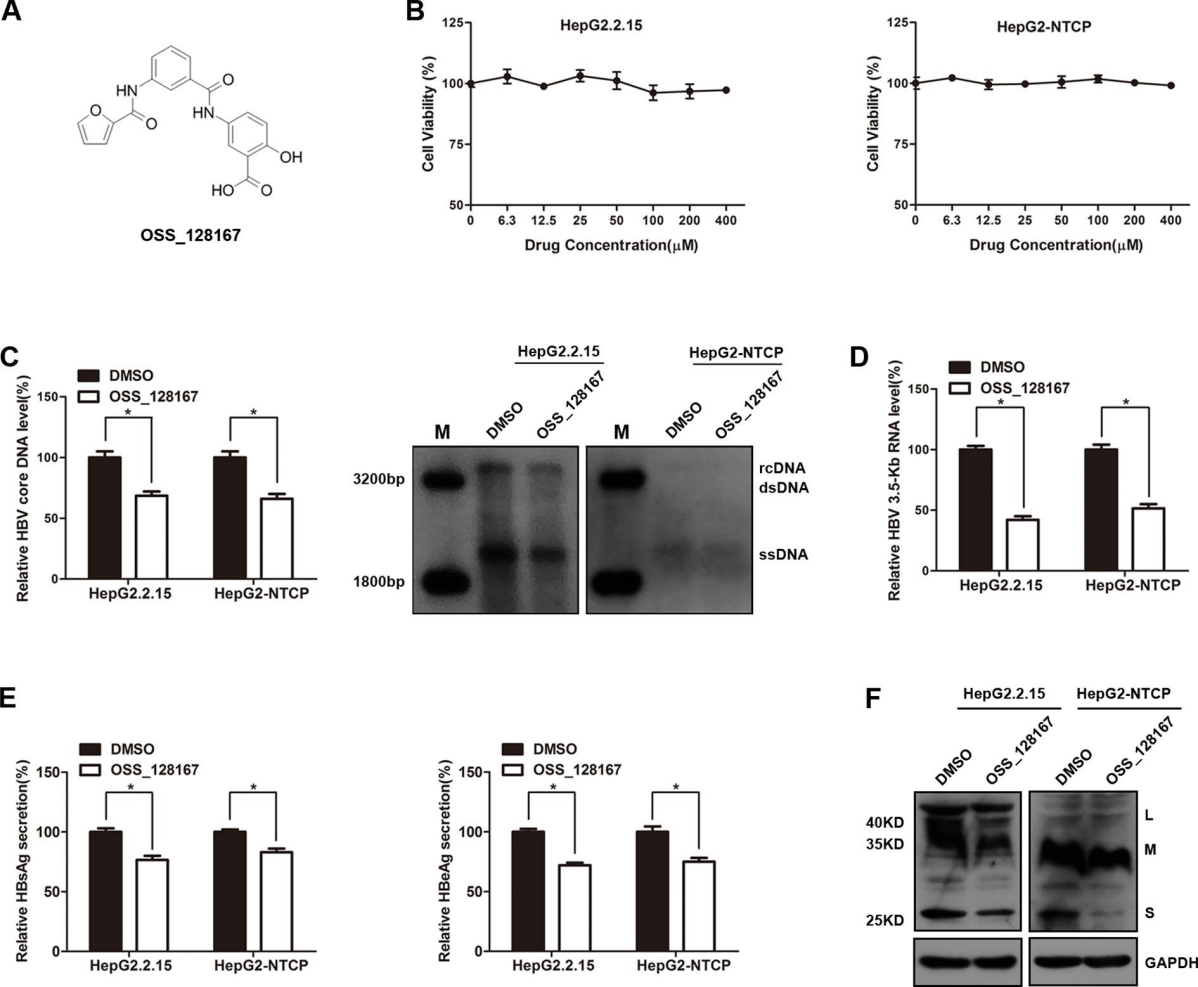
SIRT6 inhibitor OSS_128167 inhibited hepatitis B virus (HBV) transcription and replication *in vitro*. **(A)** Chemical structure of OSS_128167. **(B)** HepG2.2.15 and HepG2-sodium taurocholate cotransporting polypeptide (NTCP) cells were treated with a series of concentration of OSS_128167. 3 days post-treatment, MTS assay was performed to examine the cytotoxicity of OSS_128167. **(C**–**F)** HepG2.2.15 and HBV-infected HepG2-NTCP cells were treated with indicated concentration of OSS_128167. **(C)** Four days later, cells were harvested to examine HBV core deoxyribonucleic acid level by using real-time PCR and southern blotting analysis. **(D)** 3.5-Kb ribonucleic acid level was subjected to real-time polymerase chain reaction 3 days after OSS_128167 treatment. **(E**–**F)** Secretion of hepatitis B surface antigen (HBsAg) and hepatitis B envelope antigen were assayed by using ELISA 3 days after treatment. At the same time, HBsAg production in cell lysates was determined by western blotting. Glyceraldehyde 3-phosphate dehydrogenase was used as the loading control. Data represented the mean ± SD of three independent experiments. *:P < 0.05.

We firstly explored the potential antiviral effect of OSS-128167 in HBV stable expressing cells HepG2.2.15 or HBV-infected HepG2-NTCP cells. The chemical structure of OSS_128167 was shown in **Figure 1A**. The HepG2.2.15 and HepG2-NTCP cells were treated with a series concentration of OSS_128167 for 3 days to assess the cytotoxicity. MTS assay showed that OSS_128167 had no cytotoxicity on both two cells within 400 µM (**Figure 1B**) and 100 uM OSS_128167 was chosen for further study. Real-time PCR results revealed that OSS_128167 significantly decreased HBV core DNA level, as confirmed by southern blotting analysis (**Figure 1C**). The level of 3.5-Kb RNA was also modestly decreased after treated with OSS_128167 (**Figure 1D**). Similar to its effect on HBV core DNA and 3.5-Kb RNA levels, OSS_128167 treatment also inhibited hepatitis B surface antigen (HBsAg) and hepatitis B envelope antigen (HBeAg) secretions, as well as HBsAg expression in cell lysates (**Figures 1E, F**). These results above implied that SIRT6 inhibitor OSS_128167 might serve as a potential drug for HBV therapeutics.

### Antiviral Activity of OSS_128167 in Hepatitis B Virus Transgenic Mouse Model

**Figure 2:**
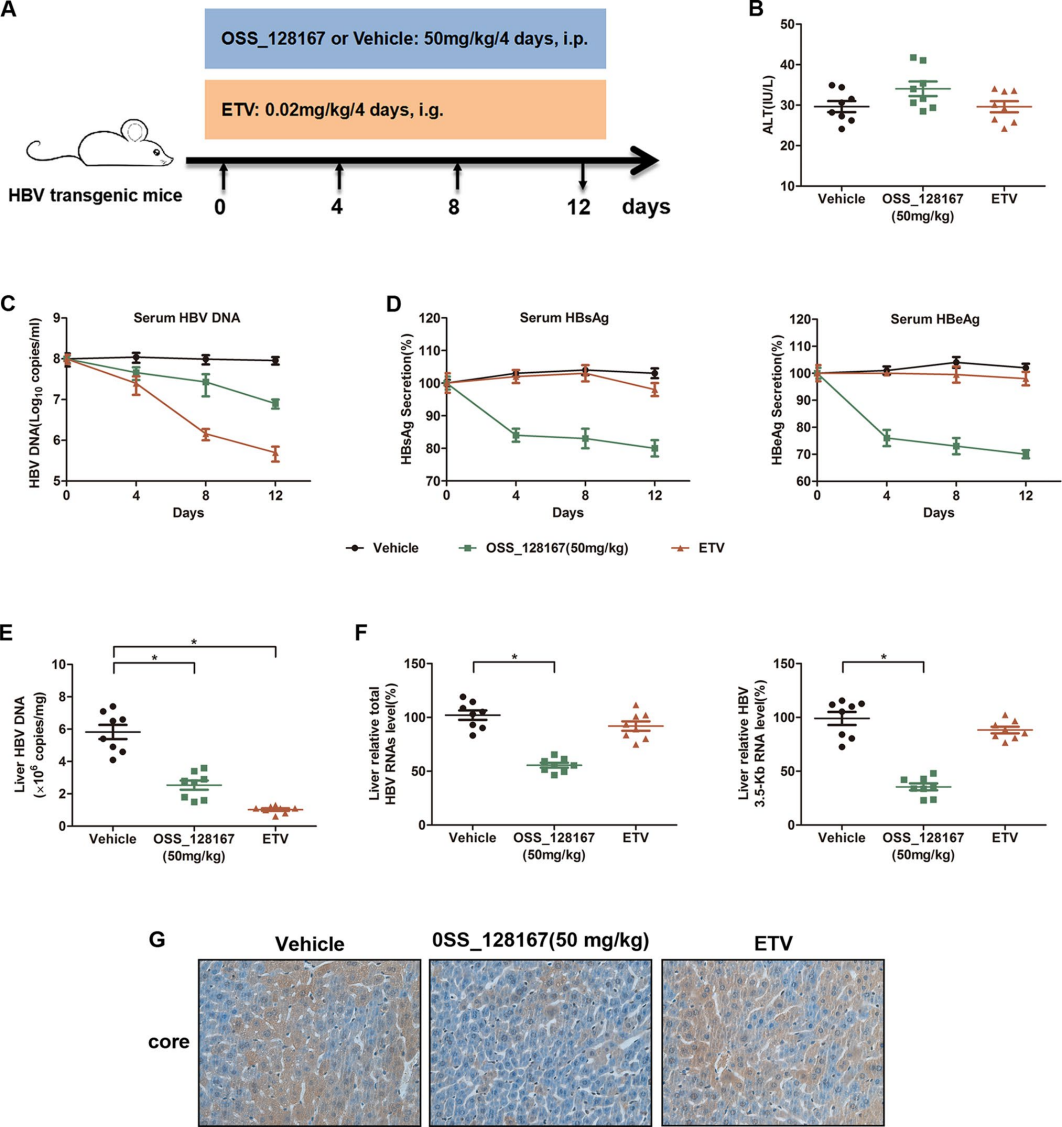
Antiviral activity of OSS_128167 in a hepatitis B virus (HBV) transgenic mouse model. **(A)** Workflow of mice model. The mice were randomly divided into three groups (n = 8). The group of OSS_128167 (50 mg/kg) and vehicle were treated every 4 days by intraperitoneal injection. The group of ETV (0.02 mg/kg) was treated every 4 days by oral gavage administration. At the indicated time points, the serum was collected through tail vein. And the mice were euthanized to collect liver tissues at 12 days post-injection. **(B)** Serum alanine aminotransferase level was determined by colorimetric microplate assay. **(C**–**D)** Kinetics of serum hepatitis B surface antigen (HBsAg), hepatitis B envelope antigen (HBeAg), and HBV deoxyribonucleic acid (DNA) were monitored during the treatment. The levels of serum HBsAg and HBeAg were assayed by ELISA. Serum HBV DNA was extracted with Viral Genome DNA/Ribonucleic Acid (RNA) Extraction Kit and determined by using real-time polymerase chain reaction (PCR). **(E)** Intrahepatic HBV DNA was extracted with Genomic DNA Extraction Kit and detected by using real-time PCR. **(F)** Liver tissues were harvested to extract total RNAs. Real-time PCR analysis was used to examine total HBV RNAs and 3.5-Kb RNA. β-Actin was used as the internal control. **(G)** HBcAg in liver tissues was detected by using Immunohistochemical staining. Data represented the mean ± SD of three independent experiments. *:P < 0.05.

To investigate whether OSS_128167 could inhibit HBV transcription and replication *in vivo*, HBV transgenic mice were administrated with 50 mg/kg OSS_128167 and the antiviral effect were analyzed. The workflow of mice model was shown in **Figure 2A**. Firstly, we examined the liver injury marker alanine aminotransferase (ALT) in different groups. Although the serum ALT was slightly increased in OSS_128167 group compared with vehicle or ETV group, there was no statistical significance (**Figure 2B**). As OSS_128167 showed no obvious hepatotoxicity, we next detected serum viral markers during the treatment and ETV, an antiviral drug approved by FDA, was used as a positive control. Encouragingly, treatment with OSS_128167 resulted in a significant reduction of HBV DNA in serum (**Figure 2C**), as well as serum HBsAg and HBeAg (**Figure 2D**), suggesting that OSS_128167 showed strong antiviral effect. Moreover, treatment with OSS_128167 resulted in a marked reduction of intrahepatic HBV DNA, total HBV RNAs and 3.5-Kb RNA level at the end of treatment (**Figures 2E, F**). By contrast, ETV treatment had no effect on HBV RNA level. Immunohistochemical analysis also showed that OSS_128167 suppressed HBV core protein expression in liver tissues (**Figure 2G**). Taken together, these data showed that OSS_128167 could inhibit HBV transcription and replication *in vivo*, indicating OSS_128167 might serve as a new therapeutic strategy for HBV treatment.

### Sirtuin 6 Regulated Hepatitis B Virus Transcription and Replication

**Figure 3:**
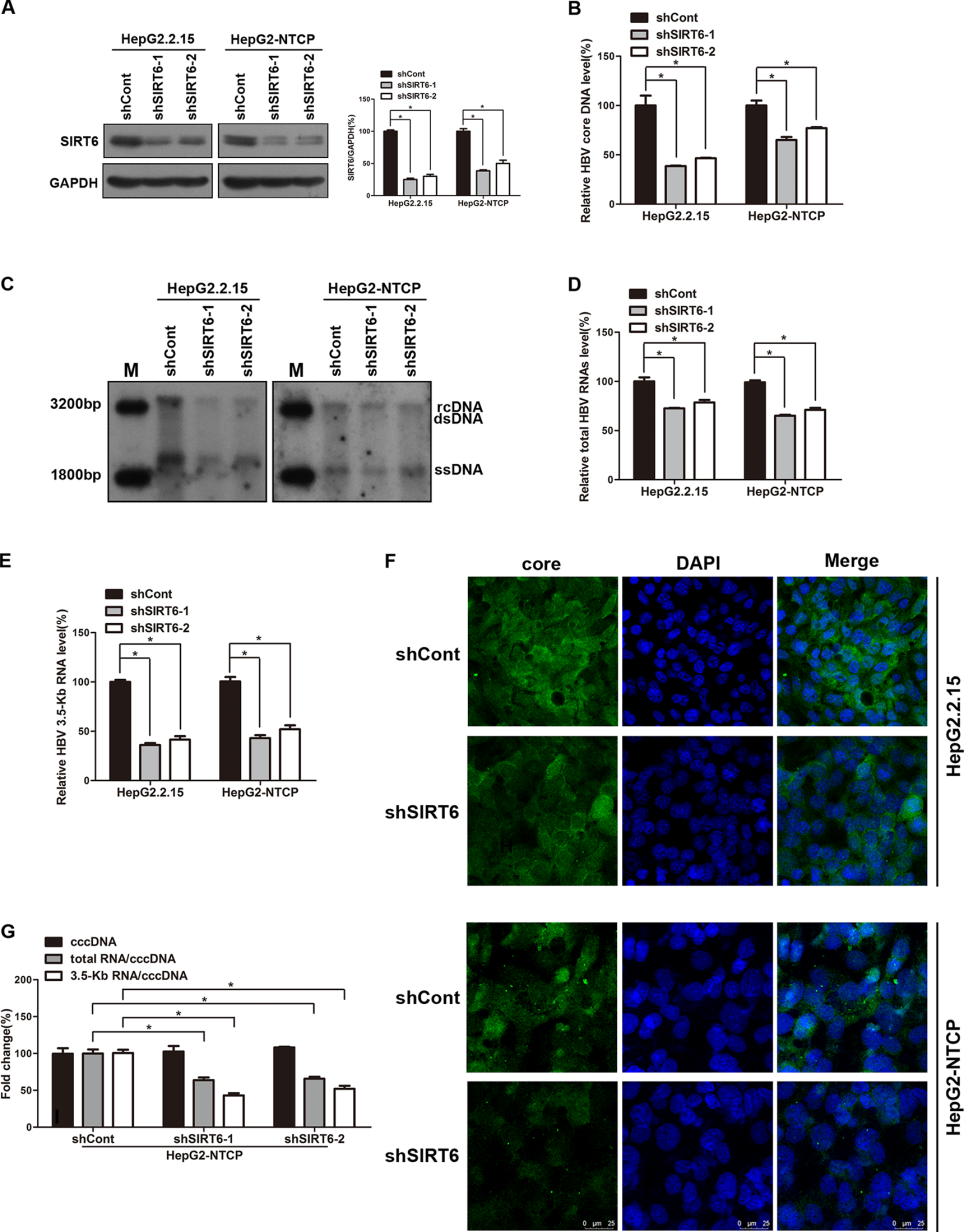
Sirtuin 6 (SIRT6) silencing restricted hepatitis B virus (HBV) transcription and replication. **(A**–**G)** HepG2.2.15 and HBV-infected HepG2-sodium taurocholate cotransporting polypeptide (NTCP) cells were transfected with plasmids expressing short hairpin ribonucleic acids (shRNAs) targeting SIRT6 (shSIRT6-1 and shSIRT6-2) or scramble control shRNA (shCont). **(A)** Total protein was extracted at 4 days post-transfection and subjected to western blotting. Glyceraldehyde 3-phosphate dehydrogenase (GAPDH) was used as the loading control. Band intensities were quantified by ImageJ software and normalized to GAPDH. **(B**, **C)** HBV core deoxyribonucleic acid (DNA) were extracted at 5 days post-transfection. Then real-time polymerase chain reaction (PCR) and southern blotting were performed to detect HBV core DNA level. **(D**,** E)** After 4 days post transfection, total RNA was extracted by using TRIzol reagent and total HBV RNAs and 3.5-Kb RNA levels were detected by real-time PCR with specific primers. β-actin was used as the internal control. **(F)** The core protein was detected by immunofluorescence staining with indicated antibody at 4 days post-transfection and the images were collected by using confocal microscope. **(G)** HBV-infected HepG2-NTCP cells were transfected with plasmids expressing shRNAs targeting SIRT6 (shSIRT6-1 and shSIRT6-2) or scramble control shRNA (shCont). HBV covalently closed circular DNA (cccDNA) was extracted and applied for real time PCR. The ratios of total HBV RNAs/cccDNA and 3.5-Kb RNA/cccDNA were calculated. Data represented the mean ± SD of three independent experiments. *:P < 0.05.

Based on the above data, we try to investigate the underling mechanism of OSS_128167 in anti-HBV transcription and replication. Considering OSS_128167 is the inhibitor of SIRT6, we firstly investigated whether SIRT6 played an important role in HBV replication process, both HBV stable expressing cells HepG2.2.15 and HBV-infected HepG2-NTCP cells were transfected with plasmids expressing short hairpin RNAs (shRNAs) targeting SIRT6 (shSIRT6-1 and shSIRT6-2) or scramble control shRNA (shCont). The silencing efficacy were firstly detected by western blotting (**Figure 3A**). Then HBV core DNA were extracted at 5 days post-transfection. In SIRT6 silencing group, the decreased HBV core DNA level was observed (**Figure 3B**), which were further confirmed by southern blotting (**Figure 3C**). In addition, SIRT6 silencing markedly reduced the level of total HBV RNAs and 3.5-Kb RNA as evidenced by real time PCR (**Figures 3D, E**). ELISA results showed a moderate decrease in HBsAg and HBeAg secretion, as well as HBsAg expression in SIRT6 knockdown cells (**Figures S1A, B**). As the main component of HBV particles, HBV core protein was examined by immunofluorescence staining. As shown in **Figure 3F**, less core protein was stained in SIRT6-silencing cells compared to control cells. Considering that HBV cccDNA is the template of all the HBV transcripts, we next explored the effect of SIRT6-silencing on HBV cccDNA in HBV-infected HepG2-NTCP cells. Our data showed that SIRT6 knockdown had no significant effect on cccDNA level (**Figure 3G**). Notably, we further tested the transcription activity of HBV cccDNA by using the ratios of total HBV RNAs/cccDNA and 3.5-Kb RNA/cccDNA. As expected, we found that the ratios were obviously reduced in SIRT6 knockdown cells (**Figure 3G**). Together, these data revealed that SIRT6 might play a functional role in regulating HBV transcription activity.

**Figure 4:**
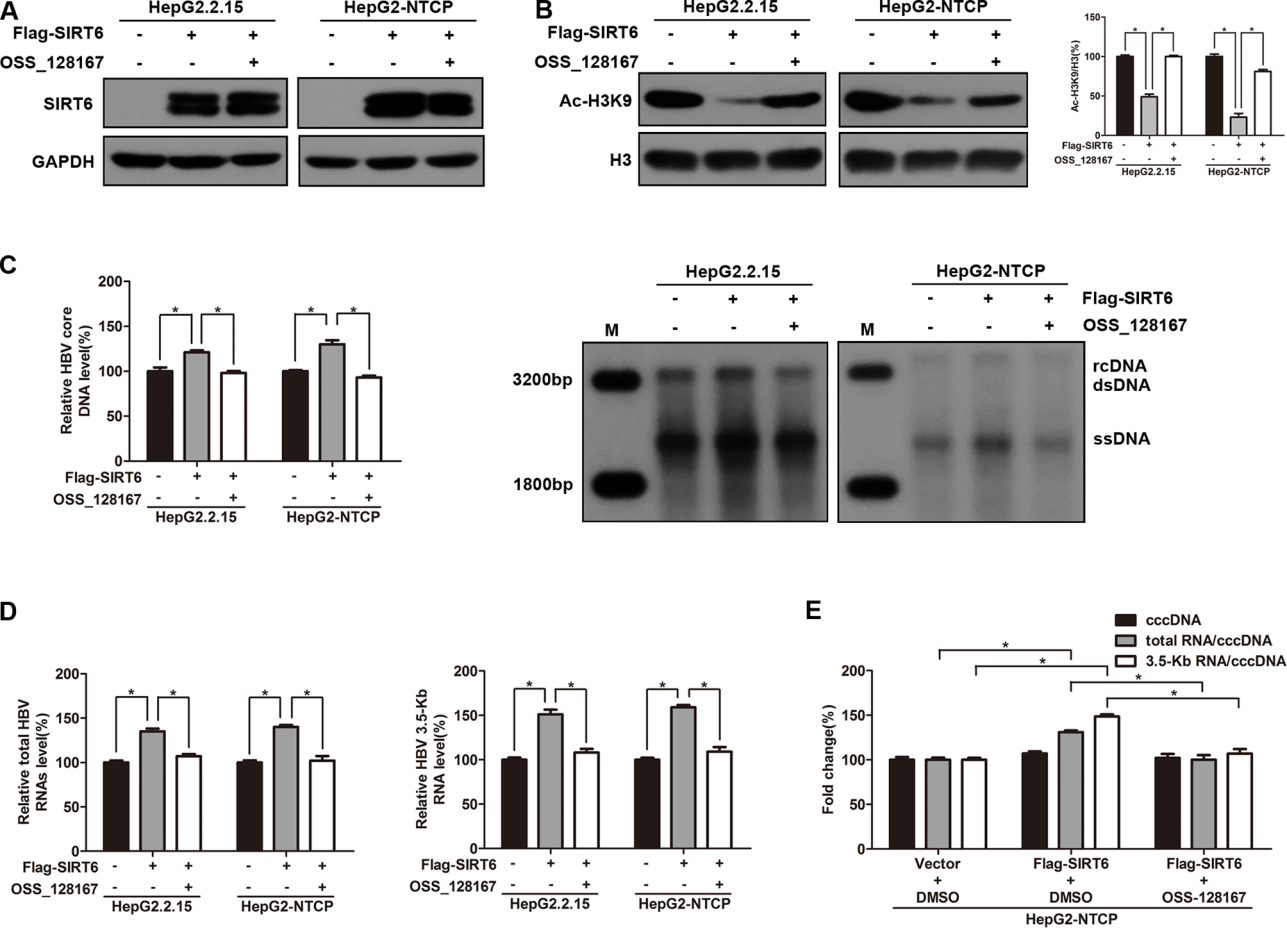
Sirtuin 6 (SIRT6) overexpression promoted hepatitis B virus (HBV) transcription depends on its deacetylase activity. **(A**–**E)** HepG2.2.15 and HBV-infected HepG2-sodium taurocholate cotransporting polypeptide (NTCP) cells were transfected with plasmids expressing Flag-SIRT6 and exposed to OSS_128167 at 1 day post-transfection. **(A**–**B)** Total protein was extracted at 3 days post-transfection and subjected to western blotting. Glyceraldehyde 3-phosphate dehydrogenase and H3 were used as the loading control, respectively. Band intensities were quantified by ImageJ software and normalized to H3. **(C)** HBV core deoxyribonucleic acid (DNA) were extracted at 4 days post-transfection. Then real-time polymerase chain reaction (PCR) and southern blotting were performed to detect HBV core DNA level. **(D)** After 3 days post-transfection, total ribonucleic acid (RNA) was extracted by using TRIzol reagent and total HBV RNAs and 3.5-Kb RNA levels were detected by real-time PCR with specific primers. β-actin was used as the internal control. **(E)** HBV-infected HepG2-NTCP cells were transfected with plasmids expressing Flag-SIRT6, then treated with OSS_128167 1 day later. HBV covalently closed circular DNA (cccDNA) was extracted and applied for real time PCR. The ratios of total HBV RNAs/cccDNA and 3.5-Kb RNA/cccDNA were calculated. Data represented the mean ± SD of three independent experiments. *:P < 0.05.

To explore the effect of SIRT6 overexpression on HBV transcription and replication, HepG2.2.15 and HBV-infected HepG2-NTCP cells were transfected with SIRT6 overexpression plasmid. Meanwhile, SIRT6 overexpressed cells were further exposed to OSS_128167 to investigate whether the functional role of SIRT6 in HBV transcription and replication could be blocked by OSS_128167. Firstly, the effect of OSS_128167 on SIRT6 expression was examined by using western blotting. Data shown that OSS_128167 had no significant effect on SIRT6 expression (**Figure 4A**). However, the acetylation status of H3K9, a known target of SIRT6 deacetylase activity, was dramatically decreased in SIRT6 overexpressed cells which could be abolished by OSS_128167 treatment (**Figure 4B**). Consistently, SIRT6 overexpression promoted HBV transcription and replication while abolished by OSS_128167 treatment, as evidenced by the level of HBV core DNA, ratios of total HBV RNAs/cccDNA and 3.5-Kb RNA/cccDNA (**Figures 4C–E**). Together, the above data indicated that the promotion of SIRT6 overexpression on HBV transcription and replication could be blocked by OSS_128167.

### Sirtuin 6 Enhanced the Activity of Hepatitis B Virus Core Promoter Through Upregulating Transcription Factor Peroxisome Proliferator-Activated Receptors Alpha

**Figure 5:**
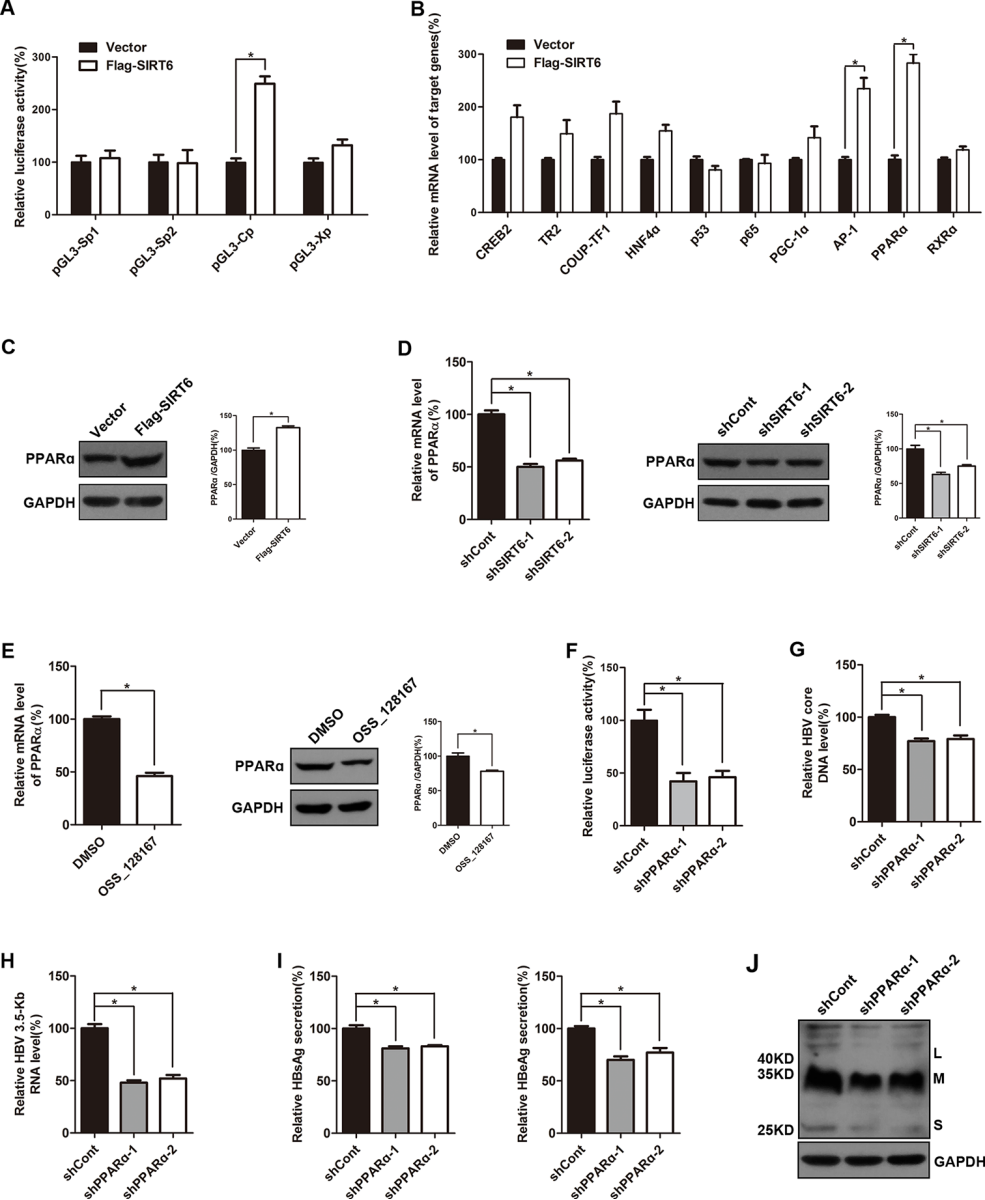
Sirtuin 6 (SIRT6) enhanced the activity of hepatitis B virus (HBV) core promoter through upregulating transcription factor peroxisome proliferator-activated receptors α (PPARα). **(A)** Four luciferase reporter constructs were cotransfected with Flag-SIRT6 into Huh7 cells, and the renilla luciferase reporter (RL-TK) was cotransfected to normalized transfection efficiency. The luciferase activities were determined 2 days after transfection. **(B)** HepG2.2.15 cells were transfected with plasmids expressing Flag-SIRT6. After 3 days post-transfection, total ribonucleic acid (RNA) was extracted by using TRIzol reagent and the expression of various transcription factors associated to HBV transcription were detected by real-time polymerase chain reaction (PCR) with specific primers. β-actin was used as the internal control. **(C)** HepG2.2.15 cells were transfected with plasmids expressing Flag-SIRT6. Total protein was extracted at 3 days post-transfection and subjected to western blotting. Glyceraldehyde 3-phosphate dehydrogenase (GAPDH) was used as the loading control. **(D)** HepG2.2.15 cells were transfected with plasmids expressing shRNAs targeting SIRT6 (shSIRT6-1 and shSIRT6-2) or scramble control short hairpin RNA (shRNA) (shCont). After 3 days post-transfection, total RNA was extracted by using TRIzol reagent and the expression of PPARα was detected by real-time PCR with specific primers. β-actin was used as the internal control. At the same time, total protein was extracted and subjected to western blotting. GAPDH was used as the loading control. Band intensities were quantified by ImageJ software and normalized to GAPDH. **(E)** HepG2.2.15 cells were treated with OSS_128167 for 3 days. The expression of PPARα was detected by real-time PCR with specific primers. β-Actin was used as the internal control. And total protein was subjected to western blotting. GAPDH was used as the loading control. Band intensities were quantified by ImageJ software and normalized to GAPDH. **(F)** The Huh7 cells were transfected with pGL3-Cp and RL-TK 24 h after transfection of plasmids expressing PPARα shRNA or scramble control shRNA. The luciferase activity was determined at 72 h posttransfection. RL-TK was used to normalized transfection efficiency. **(G**–**J)** HBV-infected HepG2-sodium taurocholate cotransporting polypeptide (NTCP) cells were transfected with plasmids expressing shRNAs targeting PPARα (shPPARα-1 and shPPARα-2) or scramble control shRNA (shCont). **(G)** HBV core deoxyribonucleic acid (DNA) were extracted at 5 days post-transfection. Then real-time PCR was performed to detect HBV core DNA level. **(H)** After 4 days post-transfection, total RNA was extracted by using TRIzol reagent and 3.5-Kb RNA level were detected by real-time PCR with specific primers. β-actin was used as the internal control. **(I**–**J)** Secretion of hepatitis B surface antigen (HBsAg) and hepatitis B envelope antigen (HBeAg) were assayed by using ELISA 4 days after transfection. At the same time, HBsAg production in cell lysates was determined by western blotting. GAPDH was used as the loading control. Data represented the mean ± SD of three independent experiments. *:P < 0.05.

To investigate the possible mechanisms of the promotion effect of SIRT6 overexpression on HBV transcription, dual luciferase reporter assay was subjected to examine the activities of HBV four promoters, including SpI, SpII, X promoter, and core promoter. Briefly, different luciferase reporter vectors which harbor different HBV promoters were cotransfected with SIRT6 and the luciferase activities were evaluated 48 h post-transfection. As showed in **Figure 5A**, SIRT6 overexpression significantly increased the activity of HBV core promoter, whereas the others had no obvious alteration, thus we focused on core promoter. As known that the activity of HBV core promoter was regulated by various transcription factors, such as RXRα, PPARα, HNF4α, p65, PGC-1α, and AP-1 (Quasdorff and Protzer, 2010). To further identify the potential transcription factors which were responsible for the SIRT6-mediated promotion of HBV transcription, cells were transfected with SIRT6 overexpression plasmids and total RNAs were extracted at 48 h post transfection. Then, core promoter related transcription factors were screened by using selective primers. Fortunately, we found that the mRNA level of PPARα and AP-1 were markedly increased in SIRT6 ectopic expression cells (**Figure 5B**). Given that the change of PPARα was more significant than AP-1, we chose PPARα for further investigation. Moreover, the effect of SIRT6 on PPARα was further checked at protein level. As expected, the protein level of PPARα increased in SIRT6 overexpressed cells (**Figure 5C**). In contrast, both mRNA and protein level of PPARα were decreased in SIRT6 knock-down cells (**Figure 5D**). And a similar result was also observed in OSS_128167 treated cells (**Figure 5E**). Taken together, the results above suggested SIRT6 might enhance HBV core promoter activity through up-regulating PPARα expression.

It has reported that PPARα could enhance the transcriptional level of HBV core promoter (Raney et al., 1997), meanwhile, upregulated PPARα was detected in our study. Thus, we examined the impact of PPARα-silencing on core promoter activity in the Huh7 cells. Undoubtedly, the activity of core promoter was inhibited by PPARα knock-down (**Figure 5F**). And the suppressive effect of PPARα silencing on HBV transcription and replication was confirmed by decrease of viral markers, such as HBV core DNA, 3.5-Kb RNA, HBsAg, and HBeAg (**Figures 5G–J**).

### Sirtuin 6 and Peroxisome Proliferator-Activated Receptors α Involved in OSS_128167-Mediated Hepatitis B Virus Transcriptional Repression

**Figure 6:**
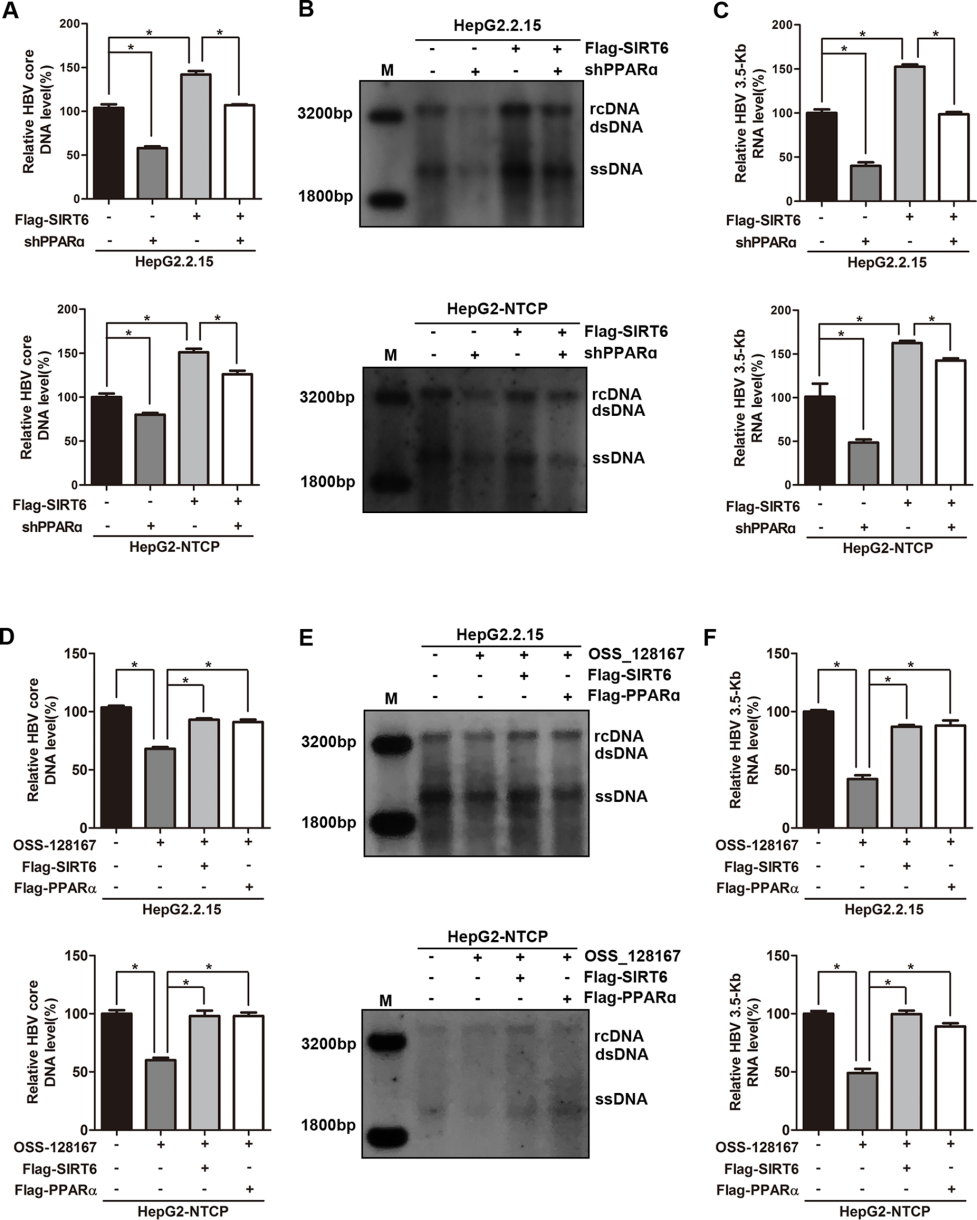
Sirtuin 6 (SIRT6) and peroxisome proliferator-activated receptors α (PPARα) involved in OSS_128167-mediated hepatitis B virus (HBV) transcriptional repression. **(A**–**C)** HepG2.2.15 and HBV-infected HepG2-sodium taurocholate cotransporting polypeptide (NTCP) cells were transfected with plasmids expressing Flag-SIRT6 1 day after transfection of construct expressing PPARα short hairpin ribonucleic acid (shRNA) or scramble control shRNA. The HBV core deoxyribonucleic acid (DNA) level was detected by real-time polymerase chain reaction (PCR) and southern blotting analysis at 4 days posttransfection of Flag-SIRT6. The 3.5-Kb RNA level was determined by real-time PCR analysis at 3 days posttransfection of Flag-SIRT6. β-Actin was used as the internal control. **(D**–**F)** HepG2.2.15 and HBV-infected HepG2-NTCP cells were respectively transfected with plasmids Flag-SIRT6 or Flag-PPARα 1 day before OSS_128167 treatment. The HBV core DNA level was detected by real-time PCR and southern blotting analysis at 4 days posttransfection. The 3.5-Kb RNA level was determined by real-time PCR analysis at 3 days posttransfection. β-Actin was used as the internal control. Data represented the mean ± SD of three independent experiments. *:P < 0.05.

To thoroughly reflect the anti-HBV process of OSS_128167, some rescue assays were performed to confirm the relationship between OSS_128167, SIRT6, PPARα, and HBV core promoter activity. Firstly, we investigated whether PPARα involved in the up-regulation of HBV transcription mediated by SIRT6. Real-time PCR and southern blotting analysis showed that PPARα-silencing abolished the increase of HBV core DNA level triggered by SIRT6 (**Figures 6A, B**). At the same time, PPARα-silencing also abolished the enhancement of 3.5-Kb RNA level induced by SIRT6 (**Figure 6C**), suggesting that SIRT6 enhanced HBV transcription and replication *via* PPARα. Subsequently, SIRT6 overexpression remarkably impaired the antiviral abilities mediated by OSS_128167 treatment (**Figures 6D–F**), demonstrating that SIRT6 involved in OSS_128167 mediated antiviral effects. In addition, PPARα also contributed to OSS_128167 mediated downregulation of HBV transcription and replication, which was proved by the restore in HBV core DNA and 3.5-Kb RNA level after PPARα overexpression (**Figures 6D–F**).

## Discussion

We estimated the antiviral effect of OSS_128167, a specific inhibitor for SIRT6, both *in vitro* and *in vivo*. Significantly, the data revealed that OSS_128167 inhibited HBV transcription and replication which shed a light that OSS_128167 might be the new potential anti-HBV drug. A number of studies has reported that SIRT6 could participate in several inflammatory signal pathways. For instance, Xiao et al. reported that SIRT6 deacetylated histone H3 lysine 9 (H3K9) at the promoter of proinflammatory genes associated with c-JUN signaling pathway (Xiao et al., 2012). Additionally, the expression of MCP-1, IL-6, and TNFɑ were increased in SIRT6-deficient macrophages. Another study showed that SIRT6 could inhibit the NF-κB activity through deacetylating the histone H3K9 at the promoters of NF-κB dependent genes, which is associated with a series of inflammatory responses (Zhang et al., 2016). Therefore, therapeutic effect of OSS_128167 in HBV infection may be also related with the elevation of antiviral immunity in hepatocytes and further studies are needed to explore the underling mechanism.

SIRT6 is the member of silent information regulator 2 (Sir2) family, which all have NAD^+^-dependent deacetylase activities. The family contains seven proteins, including SIRT1–SIRT7 (Costa-Machado and Fernandez-Marcos, 2019). Sir2 plays various role in multiple physiological and pathological process (Finkel et al., 2009). As for virus, the role of Sir2 family members in HBV replication varies from one member to another. For instance, SIRT1 and SIRT2 could enhance HBV transcription and replication (Ren et al., 2014; Cheng et al., 2018), while SIRT3 could restrict HBV transcription from cccDNA epigenetically (Ren et al., 2018). SIRT6, the member of Sir2 family, localizes in nucleus and has both deacetylase activity and mono-ADP-ribosyl transferase activity, which play an important role in regulation of DNA repair (Tian et al., 2019), telomere maintenance (Gao et al., 2018), glucose and lipid metabolism (Kuang et al., 2018), inflammation (Mendes et al., 2017). Importantly, our previous study has found that SIRT6 played a positive role in the development of HCC by enhancing tumor growth and inhibiting apoptosis (Ran et al., 2016), suggesting the pivotal role of SIRT6 in liver disease. However, the functional role of SIRT6 in HBV transcription and replication still remains to be elucidated. This study thoroughly investigated the functional role of SIRT6 in HBV infection, which is the major cause of HCC (Shlomai et al., 2014; Zhang et al., 2017). Because it is generally believed that a decrease of HBV DNA indicated the success of antiviral treatment in clinical, we evaluated the suppression effect of SIRT6-depletion on HBV DNA *in vitro*. Although multiple studies has elucidated that SIRT6 is involved in the aging-associated pathologies including metabolic disease and cancer (Liao and Kennedy, 2016; Tasselli et al., 2017), we firstly explored and announced the potential role of SIRT6 in virus replication.

An important question to address next is the underlining mechanism of decreased HBV transcription and replication regulated by SIRT6 suppression. One study displayed that SIRT6 could participate in pathological process by promoting NRF2 gene transcription (Rezazadeh et al., 2019). As for virus, only one study reported that SIRT6 played a role in inflammatory response induced by dengue virus *via* RIG-I-like receptor (RLR) and toll-like receptor 3 (TLR3) signaling pathways (Li et al., 2018), not involved the virus transcription. While, our results showed that SIRT6 could increase the transcription level of HBV cccDNA which was benefit to HBV transcription and replication. More importantly, we studied the mechanism deeply. Our data illustrated that SIRT6 could promote HBV transcription and replication by targeting core promoter. It is well known that efficient transcription of HBV core promoter is essential for 3.5-Kb RNA synthesis and cccDNA accumulation (Ko et al., 2017). Identifying the transcription factors targeting core promoter is critical to elucidate the interaction between SIRT6 and HBV. Peroxisome proliferator-activated receptors (PPAR) are a group of nuclear hormone receptor proteins, and play essential roles in the regulation of cellular differentiation (Blitek and Szymanska, 2019), carbohydrate, lipid and protein metabolism (Kersten and Stienstra, 2017). Raney et al. found that PPARα-RXRα heterodimers could interact with core promoter region spanning nucleotides −34 to −7 to enhance the activity of core promoter (Raney et al., 1997). Consistent with this finding, we also confirmed that PPARα knockdown markedly decreased core promoter activity, and HBV transcription and replication subsequently. The findings above implied that activation of PPARα might be responsible for the enhancement of HBV transcription mediated by SIRT6, and the effect of SIRT6 overexpression on HBV transcription could be blocked by OSS_128167 treatment.

In summary, we have screened and characterized the functional role of SIRT6 inhibitor, OSS_128167 in HBV transcription and replication. Mechanically, we profiled the constitutive expression of core promoter-related transcriptional factors and unfolded a role of PPARα in promoting cccDNA transcription. This study enhances our understandings of the mechanism in which host factors participate in HBV infection process and suggests that SIRT6 inhibitor, OSS_128167 may serve as a potential therapeutic application for eliminating HBV.

## Data Availability Statement

The data that support the findings of this study are available from the authors upon reasonable request.

## Ethics Statement

The animal studies were in accordance with Chinese Council on Animal Care and approved by Chongqing Medical University.

## Author Contributions

JC conducted the experimental design. JC and S-TC drafted the manuscript. HJ, S-TC, and J-HR performed most of the experiments and analyzed the experimental data. HJ and FR organized and helped to perform MTS and animal assays. S-TC, QW, and H-BY contributed to conduct western blotting analysis and southern blotting assay. A-LH and JC helped to compute and analyze the experimental data. All authors contributed to the interpretation of the data, revised the manuscript critically, and approved the final manuscript.

## Funding

This study was supported by the National Natural Science Foundation of China (81861168035, 81871656 and 81922011, JC), the Chongqing Natural Science Foundation (cstc2018jcyjAX0114, JC) and Creative Research Group of CQ University (CXQT19016, JC).

## Conflict of Interest

The authors declare that the research was conducted in the absence of any commercial or financial relationships that could be construed as a potential conflict of interest.

## Supplementary Material

Figure S1 SIRT6 silencing inhibited the secretion of HBsAg and HBeAg, as well as the production of HBsAg in cell lysates. (A-B) HepG2.2.15 and HBV-infected HepG2-NTCP cells were transfected with plasmids expressing shRNAs targeting SIRT6 (shSIRT6-1 and shSIRT6-2) or scramble control shRNA (shCont). Secretion of HBsAg and HBeAg antigen were assayed by using ELISA 4 days after transfection. At the same time, HBsAg production in cell lysates was determined by western blotting. GAPDH was used as the loading control. Data represented the mean±SD of three independent experiments. *P<0.05.
