# Supplementary figures and images for: SIRT6 Inhibitor, OSS_128167 Restricts Hepatitis B Virus Transcription and Replication Through Targeting Transcription Factor Peroxisome Proliferator-Activated Receptors α
Source: Front Pharmacol. 2019 Oct 25;10:1270. doi: 10.3389/fphar.2019.01270 (PMC6823301; doi:10.3389/fphar.2019.01270)

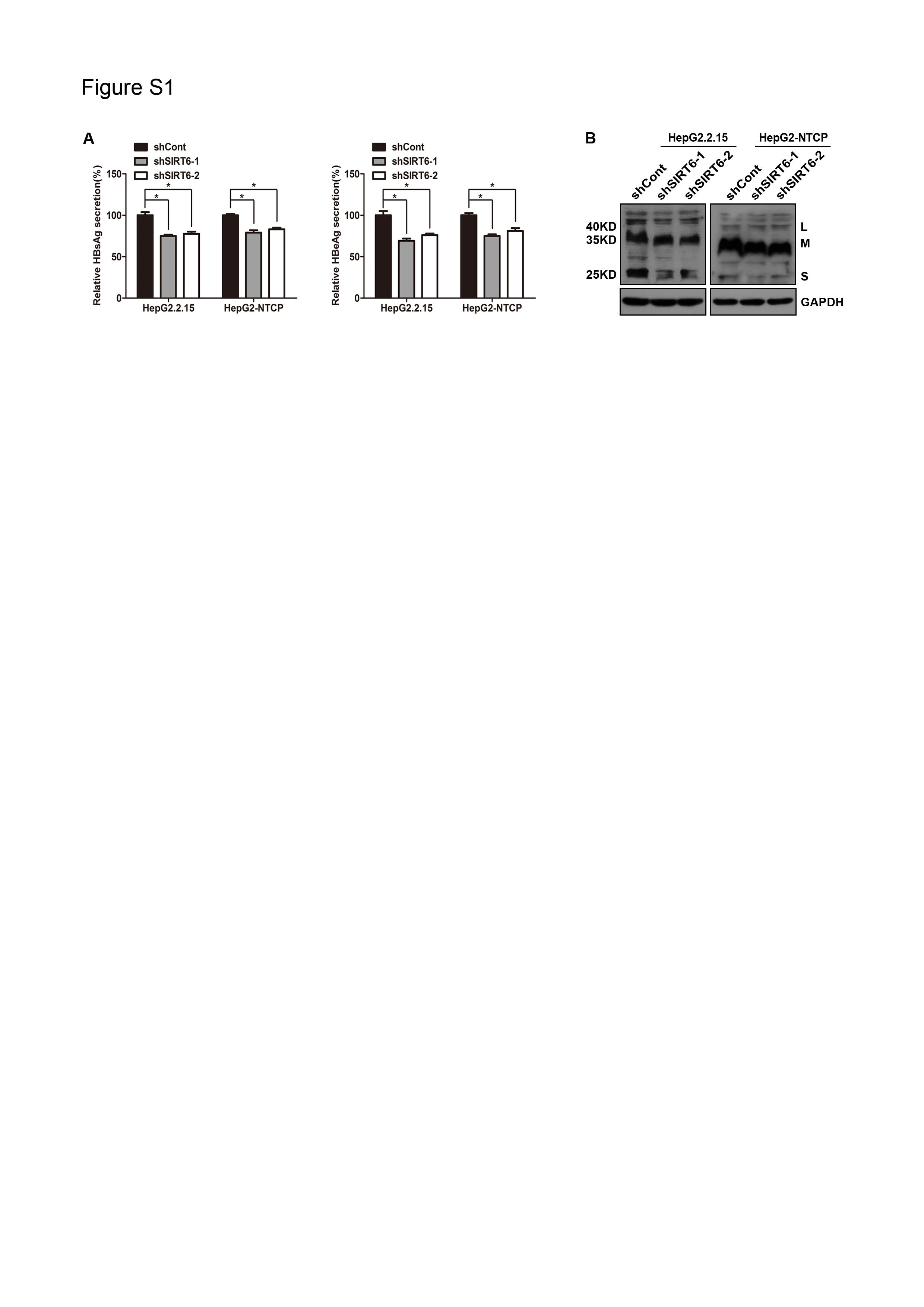

Supplement: Figure S1 — SIRT6 silencing inhibited the secretion of HBsAg and HBeAg, as well as the production of HBsAg in cell lysates. (A-B) HepG2.2.15 and HBV-infected HepG2-NTCP cells were transfected with plasmids expressing shRNAs targeting SIRT6 (shSIRT6-1 and shSIRT6-2) or scramble control shRNA (shCont). Secretion of HBsAg and HBeAg antigen were assayed by using ELISA 4 days after transfection. At the same time, HBsAg production in cell lysates was determined by western blotting. GAPDH was used as the loading control. Data represented the mean±SD of three independent experiments. *P<0.05. [file Image_1.tif]
